# Advances in Permanent Deformation Modeling of Asphalt Concrete—A Review

**DOI:** 10.3390/ma15103480

**Published:** 2022-05-12

**Authors:** Mequanent Mulugeta Alamnie, Ephrem Taddesse, Inge Hoff

**Affiliations:** 1 Department of Engineering Science, University of Agder, 4879 Grimstad, Norway; ephrem.taddesse@uia.no; 2 Department of Civil and Environmental Engineering, Norwegian Science and Technology University (NTNU), 7491 Trondheim, Norway; inge.hoff@ntnu.no

**Keywords:** permanent deformation, mechanistic, viscoplastic, viscodamage, microstructure

## Abstract

Permanent deformation is one of the dominant asphalt concrete damages. Significant progress has been made to realistically predict the damage. In the last decade, the mechanistic approach has been the focus of research, and the fundamental theories of viscoelasticity, viscoplasticity, continuum mechanics, and micromechanics are applied to develop the material laws (constitutive equations). This paper reviews the advancement of permanent deformation models including analogical, microstructural, and continuum-based methods. Pavement analysis using the nonlinear damage approach (PANDA) is the most comprehensive and theoretically sound approach that is available in the literature. The model coupled different damages and other phenomena (such as cracking, moisture, and phenomena such as healing, aging, etc.). The anisotropic microstructure approach can be incorporated into the PANDA approach for a more realistic prediction. Moreover, the interaction of fatigue and permanent deformation is the gap that is lacking in the literature. The mechanistic approaches have the capacity to couple these damages for unified asphalt concrete damage prediction.

## 1. Introduction

A flexible pavement, the longest continuous structure, comprises an asphalt concrete layer supported by unbound compacted layers (aggregate bases and subgrade). Asphaltic materials have been used for roadway construction since the end of the nineteenth century [1]. Asphalt concrete (also called bituminous mixture or hot mix asphalt) is a complex heterogeneous and three-phase material (aggregate matrix, mastic, and the air void). Such a material’s performance depends on the mixture composition, proportion, mechanical properties, and environmental conditions. It is characterized as a viscoelastic, viscoplastic, and time- and temperature-dependent material. Due to external loads and environmental factors, different distresses or damages occur in the asphalt layer. Permanent deformation or rutting is one of the primary distresses, making pavements rough and unsafe for driving, causing hydroplaning, etc. The rutting distress was noticed as a primary asphalt performance criterion [2]. The asphalt concrete’s susceptibility to permanent deformation is linked to material attributes and climatic and loading factors [3,4,5]. Material-related factors include excessive asphalt content, fine aggregate, high natural sand percentage, rounded aggregate particles, the moisture content in the mix, or granular materials and soils. From the asphalt concrete constituent properties, the chemistry of asphalt binder is the component (if not only) that makes bituminous mixtures a complex rate-dependent, nonlinear material. This nonlinear (viscous) behavior of the binder makes the permanent deformation evolution of asphalt mixtures a nonlinear mechanism [6]. For this reason, the rheological characteristics of binder (shear modulus, G*, and viscosity, η) are used to classify deformability properties of mixtures. The Strategic Highway Research Program (SHRP) rheological parameter, G*/sin *δ* (*δ*—*the phase angle*), is the widely used criterion for rutting characterization [7]. The increased value of the criteria G*/sin *δ* leads to a reduced tendency to permanent deformation. However, the G*/sin *δ* showed limitations as it cannot predict modified binders. Thus, researchers have proposed modified rheological parameters for rutting [8]. Laukkanen et al. [8] conducted multiple stress creep-recovery (MSCR) tests on unmodified and modified binders. They concluded that the non-recoverable creep compliance parameter and accumulated strain at the end of the MSCR test showed a strong correlation and predicted mixture rutting performance compared to other rheological indicators. Meena et al. [9] investigated the rutting performance of asphalt mixture through the prediction of a resilient modulus (M_R_) based on the G*/sin *δ* rheological model. The second major constituent of asphalt concrete mixtures is the aggregate. As the primary load-carrying component, the aggregate gradation, property, angularity, texture, etc., have direct influence on the permanent deformation resistance of asphalt concrete. Research has shown that fine aggregates are better for rutting resistance [6]. Decreasing the maximum aggregate size is also good for rut resistance [10], and a fine aggregate texture is highly correlated to rutting [11]. Two theories have been assumed related to fine aggregate’s role for permanent deformation; first, fillers serve to fill the voids between aggregate particles, thereby increasing the density and strength of the compacted mixture, and secondly, the fine particles of the filler become suspended in the asphaltic binder, forming a mastic. The suspended filler particles absorb binder components, hence increasing the viscosity of the binder and, consequently, the toughness of the mixes. On the contrary, Kandhal et al. [12] reported that coarse- or fine-graded Superpave mixtures do not significantly differ in rutting resistance. In addition, the aggregate type and the chemical composition also play important roles for the creep-recovery behavior of asphalt mixtures [13]. For example, siliceous aggregate mixes show a better recovery property than do calcareous aggregates. Thus, permanent deformation is a complex phenomenon where aggregate, asphalt, and asphalt–aggregate interaction (adhesion) properties control the overall performance. These properties may change over time as a result of associated damages such as aging or moisture to the asphalt–aggregate interface and fatigue cracking. Moreover, temperature-susceptible asphalt concrete and cold weather paving, which leads to low density, are factors for permanent deformation. Other climatic factors that affect rutting are temperature, precipitation, duration, type of loading, and loading extent.

The rutting of asphalt concrete is generally related to three mechanisms. The first mechanism is related to wear rutting in the wheel path, mainly due to studded tire abrasion [14]. The second mechanism of rutting is due to the viscoplastic strain accumulation (permanent deformation) in the asphalt layer. This mechanism is caused by the densification (volume change) and shear flow at a high temperature and stress level. The third form of rutting is due to a substructural failure (subsidence) of the granular subbase, subgrade layers [15,16]. Furthermore, the development of permanent deformation is a gradual and simultaneous mechanism of densification (closing of voids), shearing (slippage due to loss of adhesion between aggregates and binder), and dilation [17] as well as the initiation and growth of micro-crack damage [18]. Again, the accumulation of permanent deformation (viscoplastic strain) within the microstructure of asphalt concrete involves three phenomena: (1) viscoplastic deformation associated with the asphalt binder, (2) rotation and slippage of aggregates (evolution of the microstructure), and (3) crack initiation and propagation (microcracks and macrocracks) [19,20,21,22]. The deformation resistance of asphalt concrete is derived from the aggregate matrix and the viscous asphalt mastic. The microstructure changes due to loading (such as air void reduction) and chemical transformation (such as aging) causes the continuous modification of aggregate matrix and asphalt mastic with time. In addition, the relaxation ability of the pavement upon load removal changes as the microstructure is continuously modified [23,24,25]. Furthermore, the growth of permanent deformation is highly dependent on the stress path and strain rate [26]. Excessive deviatoric stress and the environment cause a nonlinear, plastic, and viscous flow phenomenon where the stress–strain relationship shows strong nonlinearity [27] (especially at high strains where the linear thermo-rheological properties are not valid).

In the literature, both *permanent deformation* and *rutting* terminologies are used interchangeably. The term *“Rutting”* is used to describe the pavement surface roughness due to the vertical depression along the wheel path caused by the permanent deformation or wear in the asphalt layer. Permanent deformation is the accumulation of irrecoverable strain due to densification, shear deformation, and crack growth in asphalt concrete. In this paper, the term permanent deformation is used to refer to the plastic and viscoplastic strains (irrecoverable deformation). Therefore, rutting (RD, mm) is expressed as follows.

(1) $$\mathrm{RD}=\overset{i}{\underset{i=1}{\sum }}h_{i}\varepsilon_{vp,i}$$

where $h_{i}$ is the *i*th layer’s thickness, and $\varepsilon_{vp,i}$ is the viscoplastic/permanent strain in the *i*th layer.

## 2. Objective and Scope

**Figure 1:**
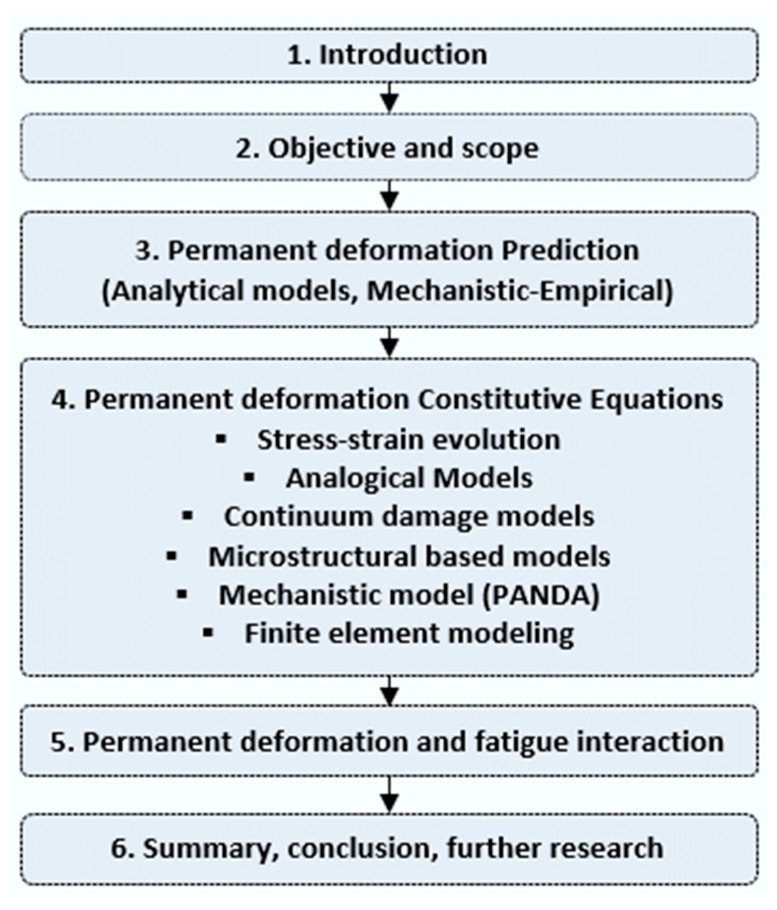
Flow chart of the study structure.

The aim of this review is to provide state-of-the art information on the developments of permanent deformation modeling for asphalt concrete. In the review, the main permanent deformation modeling theories, methods, models, and calibration tests are discussed in detail. The focus of the paper is studying the advancement of constitutive modeling approaches (analogical, microstructural, and continuum-based) and synthesize the capacity of the approaches, merits, and limitations. The robustness of permanent deformation models to account for/couple simultaneous damages such as moisture, fatigue cracking, etc., were also explored. The literature studied in this paper was collected using keywords (strings) such as viscoelastic, viscoplastic, viscodamage, permanent deformation, rutting, continuum damage, microstructure or micromechanics, mechanistic methods, creep-recovery, etc. The organization of the paper is depicted in the flow chart in Figure 1.

## 3. Permanent Deformation Prediction

### 3.1. Analytical Models

**Figure 2:**
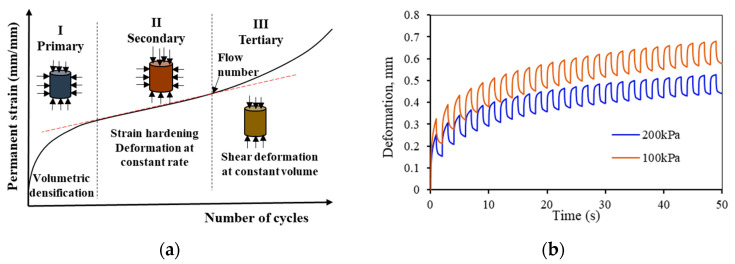
(**a**) Schematic three-stage creep deformation and (**b**) example of a creep-recovery test result at different confining pressures (100 kPa and 200 kPa).

The evolution of the irrecoverable deformation of asphaltic material due to cyclic loading is described by three distinct stages, as shown in Figure 2a. The primary zone is described by the rapid accumulation of permanent deformation at a decreasing strain rate. In the secondary zone is the constant rate of permanent deformation with strain hardening, and the tertiary stage is characterized by the increasing rate of deformation accumulate and crack formation. The flow time (FT) or the flow number (FN) is defined as the time or number of loading cycles when shear deformation under constant volume commences. Several researchers have verified that the asphalt concrete’s deformation evolution showed all the three phases [28,29,30,31,32]. This three-stage deformation property is also regarded as asphalt concrete material property. The most common and simulative test used to characterize permanent deformation is the triaxial creep-recovery test. An example in Figure 2b shows that the creep–recovery deformation is dependent on confining stress. Confinement increases the friction between aggregates and increases the resistance to deformation. Although there is no conclusive research, the in situ confining pressure of asphalt concrete is approximated to be between 100 kPa and 225 kPa.

Several analytical models have been proposed over several decades to predict the permanent deformation of asphalt concrete. Sousa et al. [33] gave a summary of selected analytical models. Some of the commonly used permanent deformation models and the corresponding calibration tests are summarized in Table 1. These analytical models are used to predict rutting from typical laboratory experimental data (axial stress–strain test data). The models in Table 1 can be classified as empirical and mechanistic-empirical, which are calibrated using simulative laboratory or field data. The models give the macroscopic responses of the measured data and hardly relate the fundamental material properties. Other models are regression equations to fit rutting data and lack the explicit physical or material property for the modeling parameters.

### 3.2. Calibration Tests

The most common laboratory test protocols used cylindrical specimens of dimensions (diameter by height) 100 mm by 150 mm for creep-recovery, or 150 mm by 50–70 mm for creep. The shear strain from simple shear tests is also used to model permanent deformation [16,34]. The coupling of shear and axial strain components in permanent deformation modeling has not been performed yet in previous studies [35]. The indirect tensile test is also used for permanent deformation with different specimen dimensions of 150 or 100 mm diameter by 50 to 70 mm thickness [36]. Moreover, the wheel tracking test is used to simulate permanent deformation [37,38,39].

**Table 1:** Some selected permanent deformation analytical models.

Model (Equation)	Variables	Description	Reference	Calibration Test
$\varepsilon_{p}={\mathrm{aN}}^{b}$	*a*, *b*	Accurate for secondary stages, small stress/strain deformation		Creep, creep-recovery
$\varepsilon_{p}={\mathrm{AN}}^{B}+C\left(e^{\mathrm{DN}}-1\right)$ Where, $A=115\left(\sigma_{1}-\sigma_{3}\right)E^{*}$ $B=0.182+0.294\left(\sigma_{\mathrm{VM}}-\sigma_{\mathrm{VL}}\right)$	*A*, *B*, *C*, *D*	Most widely used analytical model for permanent deformation in all three creep stages; the first part is a power function (for low stresses). and the second part is for high stresses (tertiary stage). ($\sigma_{\mathrm{VM}}$—maximum stress, $\sigma_{\mathrm{VL}}$– plastic failure threshold; $\sigma_{1}$, $\sigma_{3}$—axial and lateral stress, $E^{*}$—stiffness)	Francken [40]	Repeated triaxial compression
$\frac{\varepsilon_{p}}{N}={\mathrm{AN}}^{-m}$, $A=J{\left(\frac{M_{r}}{\sigma_{a}}\right)}^{-S}$	J, *S*, *m*	Analytical Power Model based on dissipated energy rate; A is a function of resilient modulus and applied stress	Khedr Safwan [41]	Multiple step dynamic test
$\varepsilon_{p}=\varepsilon_{o}e^{-{\left(\frac{\rho }{N}\right)}^{\beta }}$	$\varepsilon_{o}$, *β*, ρ	Widely used analytical model to fit all creep stages	Tseng and Lytton [42]	Triaxial creep-recovery test
$\varepsilon_{p}=\delta_{1}\left(1-e^{\delta_{2}N}\right)+\delta_{3}\left(e^{\delta_{4}N}-1\right)$	$\delta_{1}$, $\;\delta_{2}$, $\delta_{3}$, $\delta_{4}$	Analytical model for three creep stages, mainly developed for unbound materials ($\delta_{1}$, $\delta_{3}$—scale primary and tertiary strain; δ_2_, $\delta_{4}$—rate parameters)	Wilshire and Evans [43]	Creep tests
$\frac{\varepsilon_{p}}{\varepsilon_{r}}=10^{k_{1}}T^{k_{2}}N^{k_{3}}$	$k_{1}$, $\;k_{2}$, $k_{3}$	Mechanistic-empirical (MEPDG) Model $\varepsilon_{r}$—resilient strain, T—temperature		Triaxial creep-recovery test
$\varepsilon_{p}^{I}={\mathrm{aN}}_{I}^{b}$; $\varepsilon_{p}^{\mathrm{II}}=\varepsilon_{p}^{I}+c\left(N_{\mathrm{II}}-N_{I}\right)$; $\varepsilon_{p}^{\mathrm{III}}=\varepsilon_{p}^{\mathrm{II}}+d\left(e^{k\left(N-N_{\mathrm{II}}\right)}-1\right)$	*a*, *b*, *c*, *d*, *k*	Three-stage rutting model (Modified Francken model) for accurate flow number identification	Zhou et al. [28]	Triaxial creep-recovery test
$\varepsilon_{p}=A+\mathrm{BN}-{\mathrm{Ce}}^{-\mathrm{DN}}$	*A*, *B*, *C*, *D*	Two phase, Linear exponent model (mainly for unbound granular materials)	Cerni et al. [44]	Triaxial creep-recovery test
$\varepsilon_{p}=\frac{A+\mathrm{BN}}{{\left(C+N\right)}^{\alpha }}$	*A*, *B*, *C*, *α*	Incremental model: mechanistic based as a function of viscoplastic hardening (H and α), loading time, deviatoric stress, and rest period (A, C contain the parameters related to initial behavior of permanent deformation)	Choi et al. [45]	Triaxial creep-recovery
$\varepsilon_{p}=\frac{A+{\mathrm{BN}}_{red}}{{\left(C+N_{red}\right)}^{\alpha }}$ $N_{red}=N\times 10^{\alpha_{tot}}$ $\alpha_{tot}=\alpha_{\xi_{p}}+\alpha_{\sigma_{d}}$ $\alpha_{\xi_{p}}=a_{1}\xi_{p}^{a_{2}}+a_{3}$ $\alpha_{\sigma_{d}}=b_{1}{\left(\frac{\sigma_{d}}{P_{a}}\right)}^{b_{2}}+b_{3}$	$a_{1}$, $a_{2}$, $\;a_{3}$, $b_{1}$, $b_{2}$, $b_{3}$, *A*, *B*, *C*, α	A mechanistic shift model based on the load time and stress–shift function (master curve). $P_{a}$ is atmospheric pressure, $\xi_{p}$ is reduced loading time, and $\sigma_{d}$ is deviatoric stress	Choi et al. [46]	Triaxial creep-recovery
$\varepsilon_{p}=a\left(N^{b}+e^{cN}\right)$	*a*, *b*, *c*	A three-stage model modified from Francken model	Fang et al. [4]	Wheel tracking, Uniaxial cyclic compression

**Figure 3:**
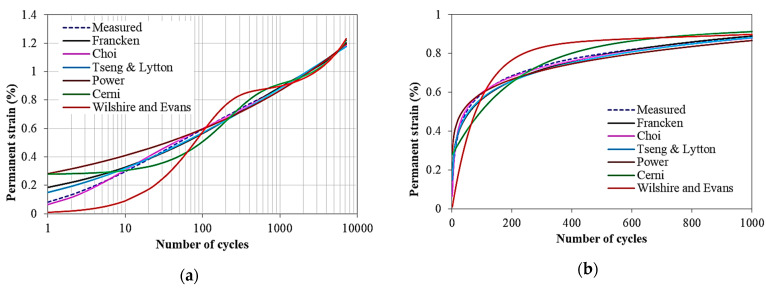
Example—permanent deformation prediction of some models using test data: (**a**) logarithmic scale and (**b**) normal scale.

As shown in Figure 3, different models have different accuracy for the same permanent deformation data. It is evident that the fitting accuracy is variable especially in the primary and tertiary stages of deformation. The incremental or Choi, Tseng–Lytton, and Francken models showed close predictions of the measured data. The Francken model is the most widely used for permanent deformation modeling [30].

Each model has a different accuracy of fitting all the creep stages of permanent deformation. Some of the models presented above have clear limitations such as the implicit empiricism, being unable to model load history and hardening–relaxation behavior, a lack of capacity to couple other simultaneous damages, etc. As discussed in the next sections, mechanistic permanent deformation prediction methods are aimed to resolve the limitations of empirical/mechanistic-empirical models. The latest mechanistic models have relied on rigorous material models, which are based on fundamental theories of mechanics, stress–strain relationships, and environmental factors.

## 4. Permanent Deformation Damage Modeling

### 4.1. Stress–Strain Response

**Figure 4:**
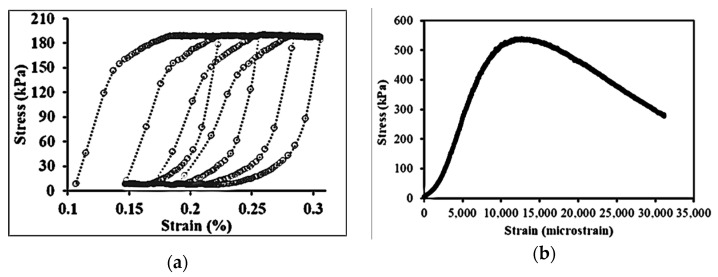
Stress–strain state in compression: (**a**) hysteresis loop under creep-recovery and (**b**) crosshead strain rate test.

The stress-, time-, and temperature-dependent viscoelastic, viscoplastic, and viscodamage properties of asphalt concrete material offered considerable challenges to accurately model the response under variable loading conditions. The response of asphalt concrete is stress path-dependent [26]. It is also a rate- and history-dependent material [47,48]. The linearity limits of asphalt concrete are 150 and 100 micro-strain in compression and tension, respectively [49], but others suggest 122 micro-strain as a limit for linear response [50]. Permanent deformation damage is induced on the asphalt beyond this strain limit at high temperatures. Moreover, the stress–strain evolution is highly dependent on the stress and strain levels, number of loading cycles (loading time), and temperature range. In a typical creep-recovery test, the stress–strain hysteresis loops evolve nonlinearly as shown in Figure 4a. The loop has the recovery and non-recovery (permanent deformation) parts. In each creep–recovery cycle, the deviatoric stress causes a non-recoverable strain and creates an open stress–strain hysteresis loop. Figure 4b shows the stress–strain responses of a constant rate compressive strength test (without recovery time). Asphalt concrete undergoes creep deformation during the load phase and a delayed recovery upon the load removal during the rest period. Traditionally, the additive decomposition of strain is applied to separate the permanent strain and recoverable strain from the total strain.

**Figure 5:**
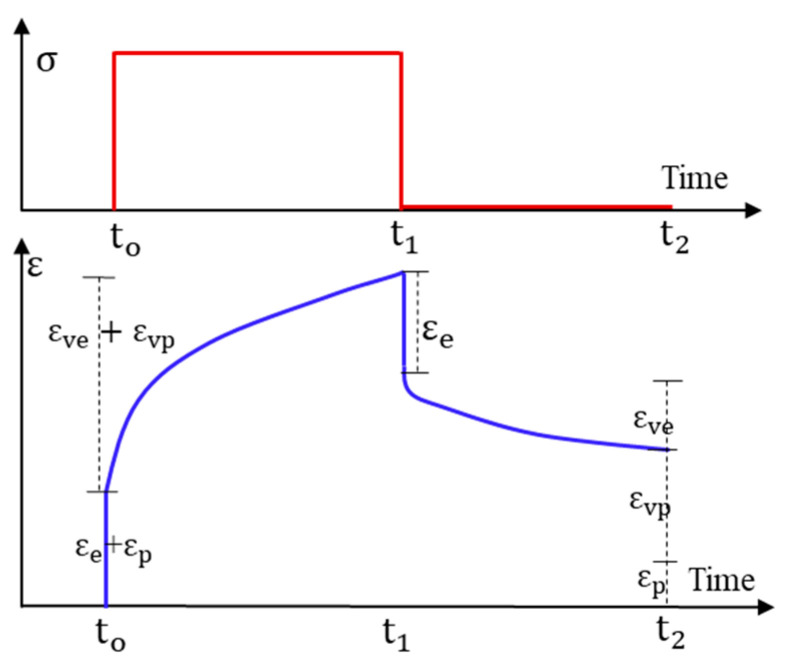
Schematic—typical creep-recovery strain components.

The schematic Figure 5 shows the strain components of a single pulse creep-recovery loading. The strain components can be separated into four strain components [51,52] (*elastic* $\varepsilon^{e}$*, viscoelastic* $\varepsilon^{\mathrm{ve}}$*, plastic* $\varepsilon^{p}$*, and viscoplastic* $\varepsilon^{\mathrm{vp}}$). The elastic (time-independent) and viscoelastic (time-dependent) are recoverable, while plastic (time-independent) and viscoplastic (time-dependent) strains are non-recoverable parts of total strain. The total strain is expressed as follows.

(2) $$\varepsilon_{\mathrm{tot}}=\varepsilon^{e}+\varepsilon^{\mathrm{ve}}+\varepsilon^{p}+\varepsilon^{\mathrm{vp}}$$

The permanent strain ($\varepsilon_{\mathrm{vp}}=\varepsilon^{p}+\varepsilon^{\mathrm{vp}}$) is obtained by subtracting the viscoelastic strain ($\varepsilon_{\mathrm{ve}}=\varepsilon^{e}+\varepsilon^{\mathrm{ve}}$) components from the total strain.

However, the strain decomposition approach is questioned when the hardening–relaxation mechanism of asphalt concrete is considered. In a loading–unloading cycle, the recoverable strain is dependent on the rest period. For tests with short rest periods, the computed viscoplastic strain can be overestimated [53]. The limitation of strain decomposition is from the inherent interaction between the viscoelastic and viscoplastic strain (the viscoelastic response is also a function of viscoplastic deformation history).

### 4.2. Constitutive Models

The computational modeling of asphalt concrete poses difficulties mainly due to the material nonlinearity, complexity to characterize under repeated and moving loads, and variable environmental conditions (temperature, moisture, etc.) [35,54]. The constitutive equations for the linear viscoelastic strain ($\varepsilon_{\mathrm{ve}}$) in undamaged conditions is defined by the Boltzmann superposition principle.

(3) $$\varepsilon_{\mathrm{ve}}\left(t\right)=\int_{0}^{t}D\left(t-\tau \right)\frac{d\sigma \left(\tau \right)}{d\tau }d\tau$$

(4) $$\sigma \left(t\right)=\int_{0}^{t}E\left(t-\tau \right)\frac{{d\varepsilon }_{\mathrm{ve}}}{d\tau }d\tau \;$$

where t is time; E(t) and D(t) are relaxation and creep compliance moduli, respectively; and τ is the integration variable. Prony series forms of the creep compliance and relaxation moduli are expressed as follows.

(5) $$D\left(t\right)=D_{o}+\overset{N}{\underset{i=1}{\sum }}D_{i}\left[1-\exp\left(-\frac{t}{\tau_{i}}\right)\right]$$

(6) $$E\left(t\right)=E_{\infty }+\overset{M}{\underset{i=1}{\sum }}E_{i}\left[\exp\left(-\frac{t}{\rho_{i}}\right)\right]$$

where N and M are the total numbers of Prony terms; $D_{o}$, $D_{i}$, and $\tau_{i}$ are creep compliance model coefficients; and $E_{\infty }$, $E_{i}$, and $\rho_{i}$ are relaxation model coefficients. Often, D(t) is obtained from E(t) data via the interconversion technique [55,56] ($\int_{0}^{t}E\left(t-\tau \right)\frac{\mathrm{dD}\left(\tau \right)}{d\tau }d\tau =1$). Once the creep compliance function is defined, the viscoelastic strain can be determined, and the viscoplastic strain is calculated from the total strain by the additive decomposition technique. A sufficient rest period is necessary to completely remove the delayed recovery strain from viscoplastic strain evolution. Cao and Kim [53] showed that 99% of viscoelastic strain is recovered for the 0.4 s pulse period and 100 s rest duration in the first cycle and 98% in cycle number 10 within 60 s. Therefore, to obtain a true viscoplastic strain, about 100 s rest period is required [45]. Experimental observations showed that the total deformation in each cycle decreases due to hardening as the number of load cycles increases. Since the viscoelastic strain is obtained from a separate dynamic modulus test (i.e., a constant viscoelastic strain), subtracting a constant cyclic viscoelastic strain from a decreasing total strain can result in a negative viscoplastic strain. That means a decreasing viscoelastic deformation model should be proposed. It is the microstructural change due to viscoplastic deformation that causes a change in viscoelastic deformation. This interaction between viscoelastic and viscoplastic deformation is referred to as *viscoelastic–viscoplastic coupling,* according to [53]. There is no available literature that couples the two deformations.

#### Analogical Models

**Figure 6:**
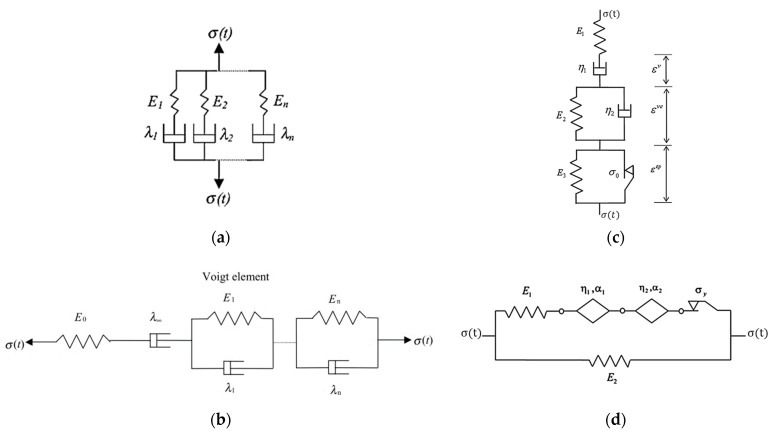
(**a**) Maxwell’s viscoelastic model, (**b**) Burgers viscoelastic model, (**c**) viscoelastic–plastic model with slider, and (**d**) elastic-visco-plastic nonlinear fractional (spring-pot) model.

The family of different analogical models has been used to model the viscoelastic–viscoplastic response of time-dependent materials [57,58,59,60]. The common classic mechanical models are spring, dashpot, slip device, pot, parabolic elements, etc., and the combination of these elements to analogs. The mechanical elements are advantageous to visualizing the stress and strain responses using the analogs. The Maxwell model (for the viscoelastic model), Kelvin model (for creep response), and the Burgers model are used to model the viscoelastic and viscoplastic strain (Figure 6a,b). The governing differential equations (viscoelastic constitutive equations) were developed from a number of springs and dashpots arranged in series and parallel. In the generalized Maxwell model, the same strain is shared across all elements, and the stress is additive, while in the generalized Burgers model the strains are additive, and the stress is the same for each element. It can be noted here that the generalized Burgers model shares the same framework as classical viscoplasticity models and allows nonlinearities based on stress to be accommodated more easily [57]. The viscoelastic and viscoplastic components can be calculated using the hereditary integral formulation as follows.

(7) $$\varepsilon_{\mathrm{ve}}\left(t\right)=D_{\mathrm{ve}}\left(0\right)\sigma \left(t\right)+\int_{0}^{t}\sigma \left(\tau \right)\frac{{\mathrm{dD}}_{\mathrm{ve}}\left(t-\tau \right)}{d\left(t-\tau \right)}d\tau$$

(8) $$\varepsilon_{\mathrm{vp}}\left(t\right)=D_{\mathrm{vp}}\left(0\right)\sigma \left(t\right)+\int_{0}^{t}\sigma \left(\tau \right)\frac{{\mathrm{dD}}_{\mathrm{vp}}\left(t-\tau \right)}{d\left(t-\tau \right)}d\tau$$

**Figure 7:**
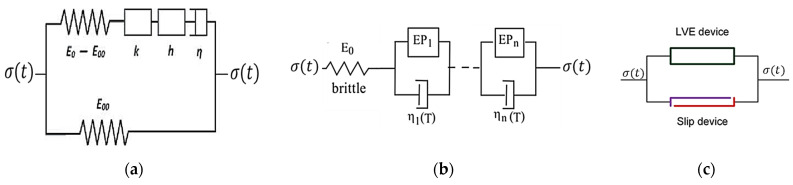
(**a**) The 2S2P1D rheological model (h and k are two parabolic creep elements), (**b**) DBN model for bituminous mixtures, and (**c**) slip device for the viscoplastic model with viscoelastic component.

$D_{\mathrm{ve}}$ and $D_{\mathrm{vp}}$ are viscoelastic and viscoplastic creep compliance. The hereditary integrals in Equations (7) and (8) are different from the one in Equation (3). A formulation based on stress and the rate of compliance rather than a formulation based on the rate of stress and compliance is advantageous to avoid problems due to the sudden application of a stress in which the rate of stress can be extremely high (e.g., in a creep test). The first derivatives of the viscoelastic and viscoplastic creep compliance for a generalized Burgers model are $\frac{{\mathrm{dD}}_{\mathrm{ve}}\left(t-\tau \right)}{d\left(t-\tau \right)}=\overset{N}{\underset{i=1}{\sum }}\frac{1}{\lambda_{i}}e^{-\left(t-\tau \right)/\tau_{i}}$ and $\frac{{\mathrm{dD}}_{\mathrm{vp}}\left(t-\tau \right)}{d\left(t-\tau \right)}=\frac{1}{\lambda_{\infty }}$, $D_{\mathrm{ve}}\left(0\right)=D_{\mathrm{vp}}\left(0\right)=0$, $\tau_{i}=\lambda_{i}/E_{i}$, where $\lambda_{i}$ is the viscosity of the *i*th Voigt element; $E_{i}$ is the modulus of elasticity; and $\lambda_{\infty }$ is the viscosity of viscoplastic element. A power function for creep compliance is also used for small stress cases. Moreover, the model in Figure 7c is a modified Burgers model with a plasticity element for asphalt mixture [60,61]. The additional elastoplastic network composed of the spring and slider in parallel is used. The limit stress in the slider modeling plasticity is denoted by $\sigma_{o}$. The authors also extended the fractional rheological model for nonlinear elastic, nonlinear viscous, and plastic properties and formulated a differential equation to characterize the viscoelastic–plastic response of asphalt concrete. Other similar analogical models such as the 2S2P1D (two springs, two parabolic. and one dashpot elements) in Figure 7a and DBN (Di Benedetto–Neifar) in Figure 7b are also frequently used to predict linear viscoelastic and creep responses (for a small number of cycles) for binders and bituminous mixtures [58,59,62]. The DBN model is a special case of the Kelvin–Voigt model where the DBN model has an elastoplastic (EP) element instead of an elastic element only. 

From these models, the governing differential equation for creep compliance or stress and strain functions is derived to predict the permanent deformation response of asphalt concrete.

**Figure 8:**
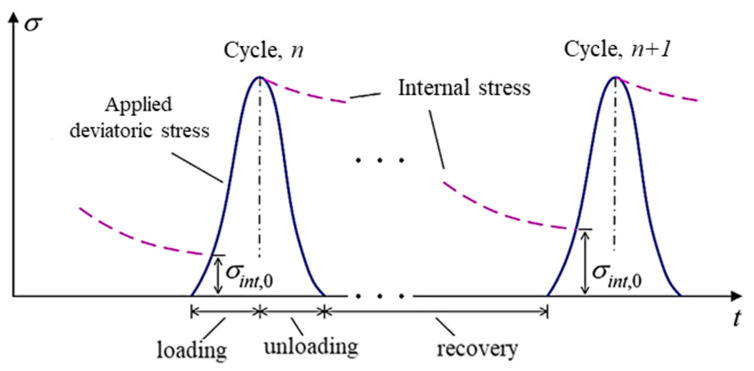
Schematic of representation of internal stress evolution during loading and unloading. Adapted with permission from [53]. 2016, Mechanics of Materials, Elsevier.

One can note that the parabolic elements (k, h) in 2S2P1D model, the slider element in (Figure 6c), and the spring-pot element in the fractional model (Figure 6d) have similar arrangements. The parabolic creep elements in 2S2P1D (Figure 7a), the elastic-plastic (EP) elements in DNB (Figure 7b) and the spring-pot elements in fractional model (Figure 6d) have also similar functions to model the elastoplastic response of asphalt concrete. The slip device shown in Figure 7c is placed in parallel with linear viscoelastic (LVE) element which has similar property as the fractional model in Figure 6c. The slip device functions as an irreversible deformation, which means that whenever the LVE device relaxes during unloading, the slip device locks, thereby disallowing strain recovery. Based on this phenomenology, the viscoelastic integrals are proposed for hardening and permanent deformation, considering that viscoelastic deformation, viscoplastic deformation, and hardening function are history dependent. Subramanian et al. [48] proposed a viscoelastic-like viscoplastic constitutive model for the permanent deformation of asphalt concrete. The proposed model takes the following form in Macaulay brackets.

(9) $$\varepsilon_{\mathrm{vp}}\left(t\right)=\langle \int_{0}^{t}D_{1}\left(t-\tau \right)\frac{{d\sigma }_{\mathrm{vp}}\left(\tau \right)}{d\tau }d\tau \rangle$$

(10) $$H\left(t\right)=H_{o}+\langle \int_{0}^{t}D_{2}\left(t-\tau \right)\frac{{d\sigma }_{H}\left(\tau \right)}{d\tau }d\tau \rangle$$

where H(t) is the material hardening variable, $H_{o}$ denotes the initial hardening state, $\sigma_{H}\left(t\right)$ and $\sigma_{\mathrm{vp}}\left(t\right)$ are functions of deviatoric stress for hardening and viscoplastic deformation calculation, and $D_{1}\left(t\right)$ and $D_{2}\left(t\right)$ are compliance functions. The two stress terms are approximated by power functions, as follows.

(11) $$\sigma_{H}\left(t\right)=H_{1}{\left(\sigma_{d}\left(t\right)\right)}^{q_{1}}$$

(12) $$\sigma_{\mathrm{vp}}\left(t\right)=\frac{{\left(G_{1}\sigma_{d}\left(t\right)\right)}^{p_{1}}+{\left(G_{2}\sigma_{d}\left(t\right)\right)}^{p_{2}}}{{\left(H\left(t\right)\right)}^{\alpha }}$$

where $\sigma_{d}\left(t\right)$ is the deviatoric stress history; and $H_{1}$, $q_{1}$, $G_{1}$, $G_{2}$, $p_{1}$, $p_{2}$, and α are parameters. This model considers only hardening during loading pulses and ignored the softening mechanism. Based on the model in [48], Cao and Kim [53] proposed a viscoplastic model using “internal stress” as the hardening variable. They hypothesized that as soon as the deformation of the LVE device is constrained (in Figure 7c), the internal stress inside the device starts to develop due to stress relaxation. As illustrated in Figure 8, the internal stress decreases with time due to LVE device relaxation before the next load cycle but has a direction opposite to the external load. Once the applied stress rises above the level of the concurrent resisting internal stress in the LVE device, the slip device is unlocked and becomes frictionless, allowing the overall deformation of the mechanical analog to increase in a viscoelastic fashion. The proposed viscoelastic-type viscoplastic model takes the following form [53].

(13) $$\varepsilon_{\mathrm{vp}}\left(t\right)=\langle \int_{0}^{t}D\left(t-\tau \right)\frac{{d\sigma }_{d}\left(\tau \right)}{d\tau }d\tau \rangle$$

This model introduces the coupling of viscoelastic and viscoplastic responses using the internal stress as a hardening variable and viscoelastic-like hardening–relaxation spectrum.

### 4.3. Continuum Based Models

#### 4.3.1. Damage Density

Continuum mechanics is a standalone and widely applied theory for damage formulation. The concept of continuum damage mechanics (CDM) was pioneered by Kachanov [63], who introduced a scalar measure called damage variable or damage density ϕ, which is defined as follows.

(14) $$\phi =1-\frac{A}{A_{d}}=\frac{A_{d}-A}{A_{d}}$$

where A—real (intact) area, $A_{d}$—damaged area, $A_{d}-A$ is the area of micro-damage, ϕ=0 means the initial state, and ϕ=1 mean complete rupture. Based on the damage density function and effective area, the effective stress concept in CDM is defined as follows.

(15) $${\bar{\sigma }}_{\mathrm{ij}}=\frac{\sigma_{\mathrm{ij}}}{1-\phi }$$

where ${\bar{\sigma }}_{\mathrm{ij}}$ is the effective stress tensor in an undamaged configuration; and $\sigma_{\mathrm{ij}}$ is the nominal Cauchy tensor in damage configuration. For more accurate modeling, the damage evolution is modified as follows [64].

(16) $${\bar{\sigma }}_{\mathrm{ij}}=\frac{\sigma_{\mathrm{ij}}}{{\left(1-\phi \right)}^{2}}$$

The classic Kachanov–Robotnov damage models [63,65] were extensively used for creep damage ($\phi_{c}$) modeling for different materials.

(17) $$\mathrm{Kachanov}:\;\dot{\phi_{c}}=G{\left(\frac{\sigma }{A}\right)}^{r}{\left(1-\phi_{c}\right)}^{-k}$$

(18) $$\mathrm{Robotnov}:\;\dot{\phi_{c}}=\frac{C_{1}\sigma^{\gamma }}{{\left(1-\phi_{c}\right)}^{\eta }},\;\dot{\phi_{c}}=\frac{C_{2}\exp\left(k\varepsilon \right)}{{\left(1-\phi_{c}\right)}^{\eta }}$$

where A, G, r, k, and $C_{1}$, γ, η, $C_{2}$, k are material constants. Recently, Darabi et al. [64] developed a continuum viscodamage model using the effective total strain ($\varepsilon_{\mathrm{ef}}^{T}$), viscoplastic hardening, and temperature coupling functions.

(19) $$\dot{\phi_{c}}=\Gamma_{o}^{\varphi }{\left[\frac{\bar{Y}{\left(1-\phi_{c}\right)}^{2}}{Y_{0}}\right]}^{q}\exp\left({k\varepsilon }_{\mathrm{ef}}^{T}\right)G\left(T\right)$$

where $\Gamma_{o}^{\varphi }$ is reference damage viscosity, $Y_{0}$ is reference damage force, $\bar{Y}$ is damage driving force in effective configuration, G(T) is temperature coupling term, and k and q are constants.

#### 4.3.2. Viscoplasticity

**Figure 9:**
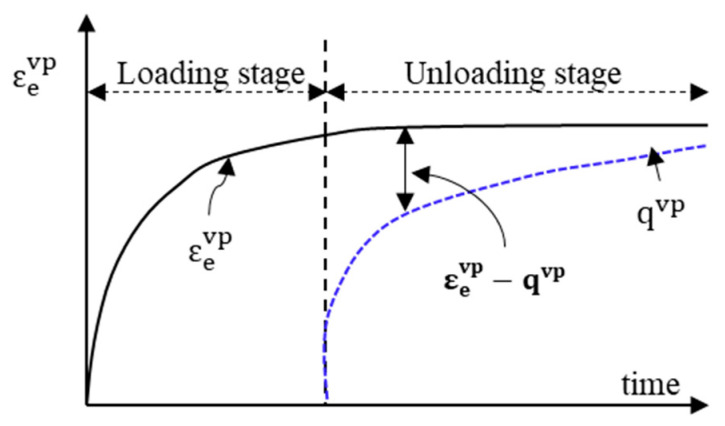
Schematic—the evolution of the effective viscoplastic strain and the hardening–relaxation during loading–unloading cycle. ($\varepsilon_{e}^{\mathrm{vp}}-q^{\mathrm{vp}}$ the driving force for viscoplastic softening or recovery in the viscoplastic hardening). Adapted with permission from [23], 2012, International Journal of Plasticity, Elsevier.

The classic Perzyna viscoplastic hardening rule [66] assumes a constant hardening variable for a cyclic creep-recovery load, defined as

(20) $${\dot{\varepsilon }}_{\mathrm{vp}}=\Gamma^{\mathrm{vp}}f\frac{\partial F\left(\sigma \right)}{\partial \sigma }$$

where $\Gamma^{\mathrm{vp}}$ is the viscoplastic fluidity parameter such that $1/\Gamma^{\mathrm{vp}}$ is a measure of viscoplastic viscosity; and $\frac{\partial F\left(\sigma \right)}{\partial \sigma }$ is a measure of the direction of viscoplastic strain. The classical hardening assumes that the viscoplastic strain rate decreases progressively with an increase in loading time. However, the hardening function is not constant due to the hardening–relaxation behavior [23]. Different researchers pointed out that the Perzyna-type rate models have limitations such as that the model cannot capture the load history effect, the relaxation or softening behavior during the rest period is ignored, and it assumes a constant hardening parameter [23,47,48]. As illustrated in Figure 9, the viscoplastic strain rate is no longer a decreasing function. The quantity q^vp^ is the hardening–relaxation internal state variable that memorizes the maximum experienced viscoplastic strain for which the hardening recovery has occurred.

### 4.4. Mechanistic Methods

#### 4.4.1. Pavement Analysis Using the Nonlinear Damage Approach (PANDA)

**Figure 10:**
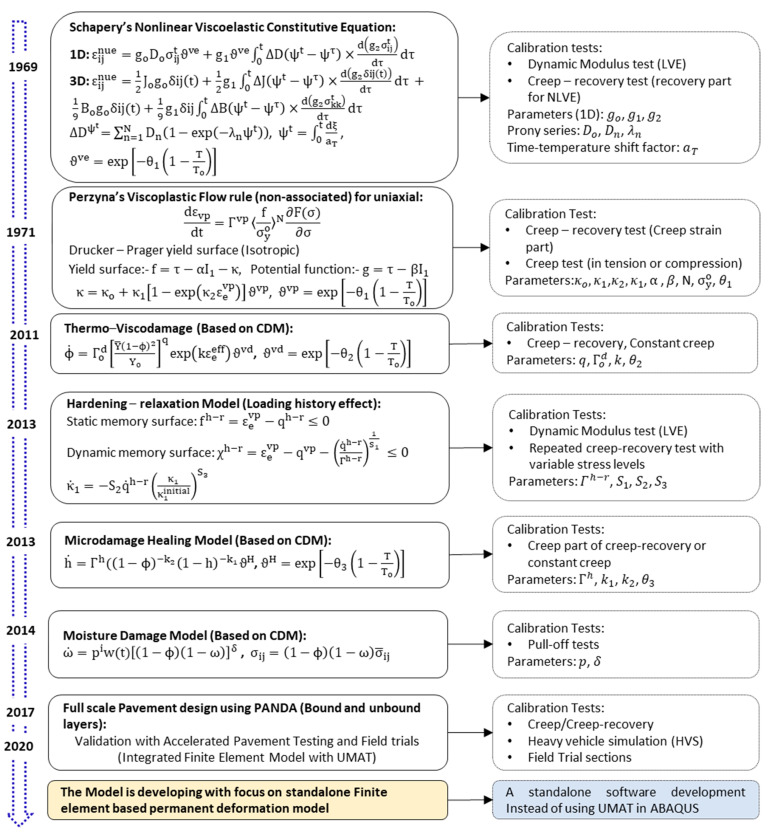
Development of the PANDA mechanistic permanent deformation model.

Pavement analysis using the nonlinear damage approach (PANDA) is the latest generation of mechanistic pavement design approach [67]. The PANDA is a mechanistic-based pavement analysis method that is founded on three classic theories: (1) Schapery’s [68] nonlinear viscoelasticity, (2) Perzyna’s [66] viscoplasticity, and (3) Darabi’s [64] viscodamage constitutive relationship. Based on the three constitutive equations, the PANDA approach has an unlimited capacity to couple different damage mechanisms of pavement structures. The approach coupled the temperature, rate, and time-dependent viscoelastic and viscoplastic models to predict the permanent deformation of asphalt concrete. For example, healing, aging, hardening–relaxation, moisture-induced damage, and other behaviors are conveniently incorporated into the PANDA approach [69,70,71,72,73]. In Figure 10**,** the development of the PANDA model and the constitutive equations are summarized.

(1) First, the linear and nonlinear viscoelastic variables are obtained from dynamic modulus (for linear viscoelastic) and creep-recovery tests (nonlinear viscoelastic). The nonlinear viscoelastic strain is formulated using the well-known Schapery’s viscoelastic constitutive equation [68].
(2) Secondly, the viscoelastic strain is deducted from the total strain to extract the viscoplastic strain from the same creep-recovery test data using the strain decomposition principle. Then, the classic Perzyna’s viscoplasticity [66] theory is adopted to predict the viscoplastic strain evolution. The Drucker–Prager yield surface function is often used [23,74].
  (21) $${\dot{\varepsilon }}_{\mathrm{vp}}=\Gamma^{\mathrm{vp}}{\langle \frac{f}{\sigma_{y}^{o}}\rangle }^{N}\frac{\partial F\left(\sigma \right)}{\partial \sigma }$$
(3) The third foundation of the PANDA constitutive model is the viscodamage model [64] using the continuum damage mechanics (CDM) theory. The effective strain is used in effective configuration. The viscodamage model mainly predicts the permanent deformation in the tertiary creep.

Therefore, the PANDA model encompasses nonlinear viscoelastic, viscoplastic (hardening), and viscodamage responses. The thermo-piezo-rheological viscoelastic properties [75] coupled with the viscoplastic yield criteria (the Drucker–Prager yield surface) is an integral part of the PANDA method [76]. Several material parameters need to be optimized to calibrate the viscoelastic–viscoplastic–viscodamage, the hardening–relaxation, moisture damage, and healing responses of asphalt concrete. The parameters and their physical meaning are presented in Table 2 Despite the unlimited capacity of the PANDA approach, calibrating the large number of model parameters is a laborious task. Hence, a systematic procedure is followed to obtain material parameters with a smaller number of tests. Once robust mechanistic constitutive models are developed and calibrated, the numerical implementation (finite element modeling) is performed using the user-defined material (UMAT) tool to define material laws in commercial software such as ABAQUS. Finite element modeling (FEM) is conducted at the desired modeling space (2D or 3D), and realistic tire–pavement contact [77], traffic, and full pavement structure (asphalt, base, subbase, and subgrade), etc., can be constructed [67,76,78,79,80]. Although the PANDA approach is still at a research stage, it is evident that advantages as well as limitations can be listed. In Table 3, some of the merits and limitations of the PANDA are described. The calibration of PANDA models used uniaxial creep, uniaxial constant stress creep-recovery, the crosshead strain rate test, and multiple stress creep tests, etc. The influence of confining pressure on the linear viscoelastic as well as nonlinear viscoelastic responses is significant [75,81]. Most studies that tried to calibrate the PANDA models used uniaxial test data, or some used a single confining pressure.

#### 4.4.2. Microstructural Based Models

**Figure 11:**
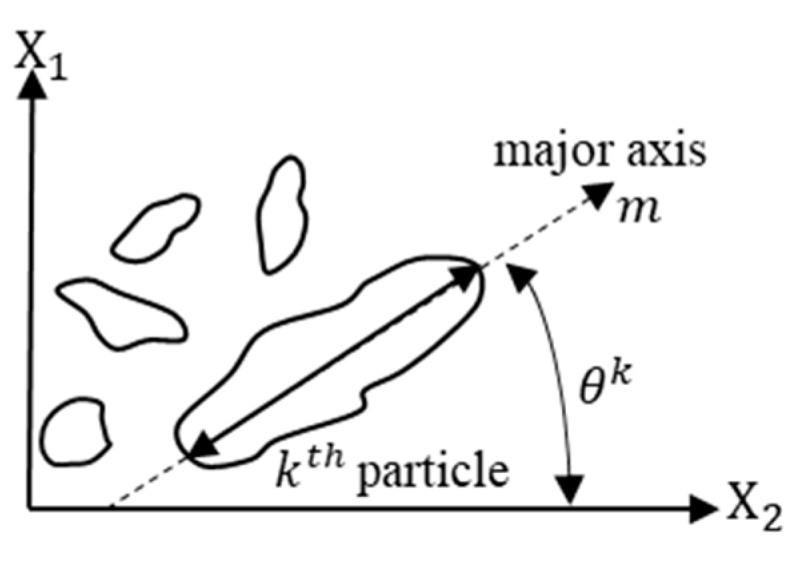
Two-dimensional particles orientation for micromechanics modeling (orientation, θ and vector, *m*).

The micromechanics approach is probably the best way to account the effects of individual mixture constituents and their interactions and the anisotropy of heterogenous asphalt mixture. The microstructure change in asphalt concrete is mainly due to the friction between the aggregates and interlocking bond breakage. This mechanism is responsible for the accumulation of permanent deformation rather than the recoverable part of the deformation. The continuous increase in the resistance of the material due to the permanent microstructure rearrangement is physically related to the strain hardening. The hardening parameter reflects the combined effect of the cohesion of asphalt binders, the adhesion properties between binder and aggregate, and the frictional properties of the aggregate structure. The fabric of granular media refers to the size, shape, and arrangement of the solid particles and the associated voids. The scalar quantity, like void ratio, is not capable of characterizing the directional nature of fabric and describing the state of packing of granular materials [85]. The microstructure approach is necessary to consider the directional nature of granular fabric. The approach is capable of modeling nonlinearities such as heterogeneity, aggregate distribution, anisotropy, crack, and air void. The micromechanics coupled with the continuum damage approach is a powerful way to model the permanent deformation of granular asphalt concrete with an appropriate representative volume element (RVE) [19,20,21,22]. The fundamental element of the granular microstructure is a directional unit vector *m* and the vector magnitude Δ (Figure 11).

(22) $$\Delta =\frac{1}{M}{\left[\overset{M}{\underset{k=1}{\sum }}{\left(\cos2\theta^{k}\right)}^{2}+\overset{M}{\underset{k=1}{\sum }}{\left(\sin2\theta^{k}\right)}^{2}\right]}^{\frac{1}{2}}\;$$

where M is the total number of objects analyzed in an image; and $\theta^{k}$ is the orientation of the unit vector n and ranges between −90 and +90°. Theoretically, the value of Δ is between zero and unity; Δ=0 indicates that objects are completely randomly distributed, which is analogous to isotropic materials; and Δ=1 indicates that objects are entirely oriented in one direction, which is analogous to perfectly transverse anisotropic materials. Oda and Nakayama [86] introduced a symmetrical microstructure tensor $F_{ij}$ that gives a measure of the two-dimensional anisotropy produced by the preferred orientation of non-spherical particles.

(23) $$F_{ij}=\left(\begin{array}{ccc} \left(1-\Delta \right)/\left(3+\Delta \right) & 0 & 0\\ 0 & \left(1+\Delta \right)/\left(3+\Delta \right) & 0\\ 0 & 0 & \left(1-\Delta \right)/\left(3+\Delta \right) \end{array}\right)$$

For simplicity the anisotropy value can be taken as constant (the initial value). For isotropic elements, $F_{11}=\left(-4\Delta \right)/3\left(3+\Delta \right)$ and $F_{22}=F_{33}=\left(2\Delta \right)/3\left(3+\Delta \right)$.

**Figure 12:**
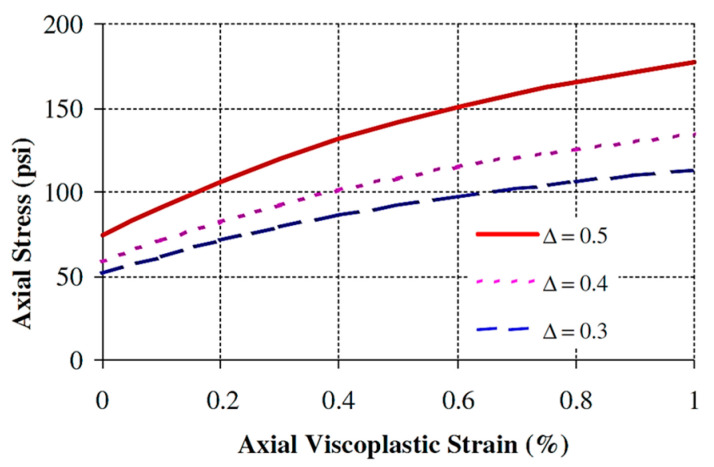
Effect of anisotropy on the stress–strain relationship predicted by the model. Reprinted with permission from [87]. 2005, International Journal of Plasticity, Elsevier.

The microstructure tensor is incorporated in the Drucker–Prager yield function by modifying the first and second invariants. The classical Perzyna’s viscoplastic and continuum damage models are then used to formulate the microstructure-based viscoplastic strain model to characterize permanent deformation of asphalt concrete. The detail derivation of the model can be found in the study [19,20,86]. The final form of the viscoplastic model in a triaxial compression test (axial strain) based on micro-structural anisotropy takes the following form.

(24) $${\dot{\varepsilon }}_{\mathrm{vp}}=\frac{\left[\sqrt{X}-\beta \left(1-Y\right)\right]}{\sqrt{\frac{3}{2}X+3\beta Y\sqrt{X}+3\beta^{2}+\frac{3}{2}\beta^{2}Y^{2}}}\sqrt{{\dot{\varepsilon }}_{\mathrm{ij}}^{\mathrm{vp}}{\dot{\varepsilon }}_{\mathrm{ij}}^{\mathrm{vp}}}={\dot{\varepsilon }}_{11}^{\mathrm{vp}}\;$$

where $X=\frac{1}{3}-\frac{4}{9}\sqrt{24}\mu {\left(\frac{\Delta }{3+\Delta }\right)}^{2}$, $Y=\frac{4}{3}\sqrt{24}\lambda {\left(\frac{\Delta }{3+\Delta }\right)}^{2}$, and λ and μ are anisotropy coefficients that reflect the effect of the aggregate anisotropic distribution on the confining and shear stresses, respectively. The viscoplastic model considers phenomena including the effect of the binder fluidity (Γ); confinement and aggregate friction (α); aggregate interlocking and dilation (β); binder cohesion and its adhesion to the aggregates (κ); anisotropy of aggregate distribution (Δ); and microstructure damage (ξ). As shown in Figure 12, the anisotropy parameter significantly contributes to compressive viscoplastic behavior and has little effect or no effect on the tensile and linear viscoelastic property [22]. In a conventional continuum method (isotropic mode), phenomenologically motivated, microstructural-based viscoplastic models have been developed, for example in [23,25] and others.

**Figure 13:**
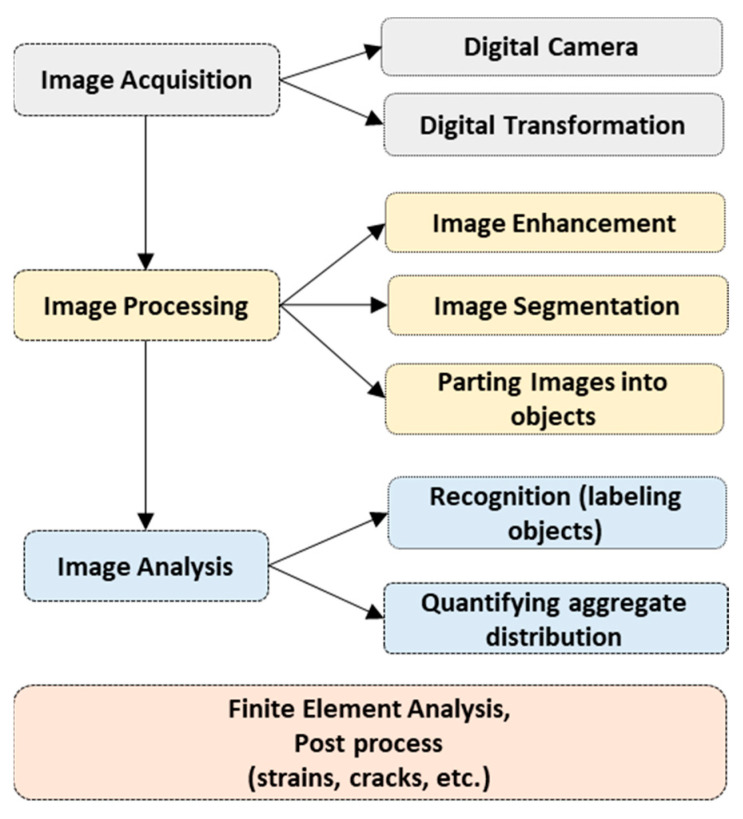
Digital image analysis process for microstructure modeling.

**Figure 14:**
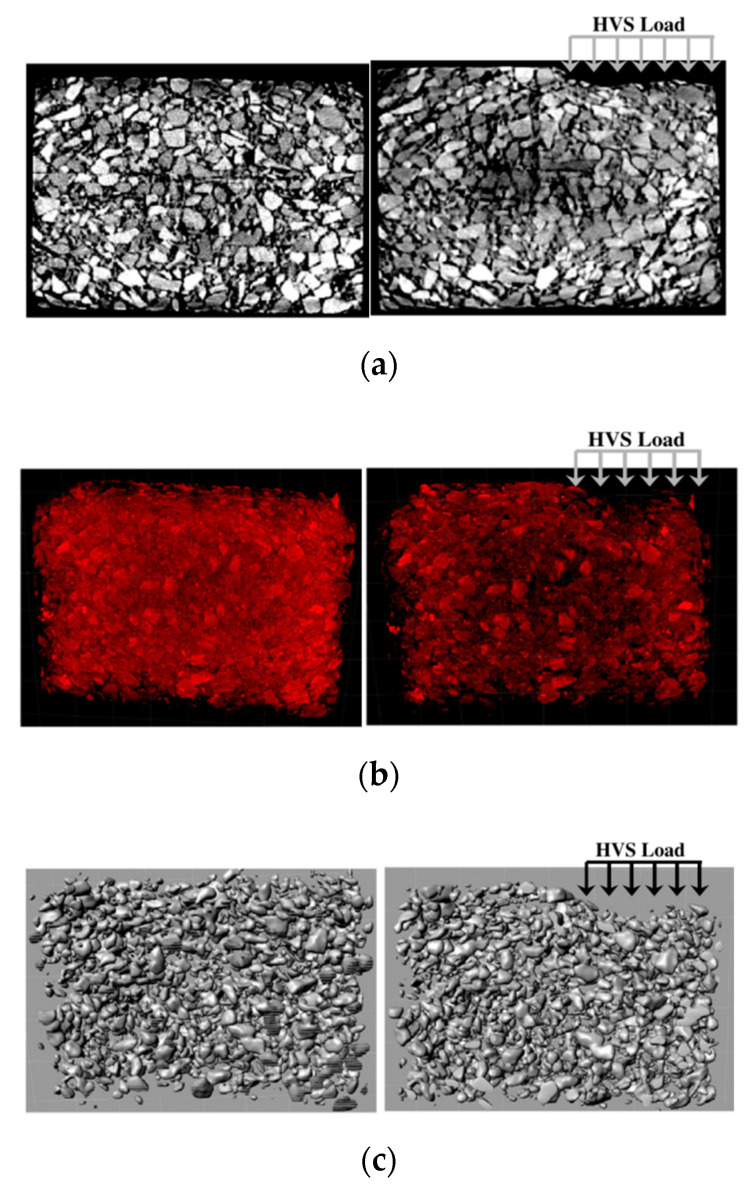
The procedure followed for aggregate domain creation: (**a**) unprocessed 2D X-ray CT image used for the development of image stacks (left: before HVS trafficking and right: after HVS trafficking), (**b**) 3D images developed from X-ray CT image stacks (left: before HVS trafficking and right: after HVS trafficking), and (**c**) 3D aggregate volumes (left: before HVS trafficking and right: after HVS trafficking). Note: direction of HVS traffic is out of the page. Reprinted with permission from [88]. 2012, Construction and Building Materials, Elsevier.

Microstructure modeling is directly integrated with the utilization of digital technologies to capture the particles arrangement, deformation, etc., in the granular materials packing. Digital image correlations, X-ray chromatography, and other tools were used for asphalt concrete. Coleri et al. [88] used the X-ray computed tomography (CT) method to model asphalt concrete rutting in a full-scale heavy vehicle simulation (HVS) test site. The object segmentation and processing procedure using a digital camera is shown in Figure 13, and an example of the discretization phases is shown in Figure 14. Using the digital technology, parts of a heterogenous mixture can be easily dissected, modeled, and analyzed into the FEM and other post processes.

In general, the microstructural based micromechanics modeling is ideal to consider the effect of individual constituent particles in viscoplastic evolution [89]. However, this approach needs image analyzing tools, discretization, and finite element modeling. Several material behaviors such as hardening–relaxation, healing, stress history, viscodamage, etc., need to be integrated for accurate formulation. Limited literature can be found in this area. With the advancement of morphological image analyzing tools, the microstructural approach will be the next active research area to characterize permanent deformation.

#### 4.4.3. Finite Element Simulation

Even if pavement is considered a homogeneous, isotropic, elastic, multi-layered system, the calculated stress–strain distribution in the structure under the simple action of a passing wheel is very complex. For a realistic modeling of pavements, each layer and material constituent should be modeled with the appropriate material constitutive law, and the interaction of each layer should be analyzed as a pavement system. The finite element method is the most versatile approach in the mechanistic pavement design approaches. The FEM is an integral part of the PANDA approach; the modeling space (2D or 3D), wheel loading configuration, constitutive law, etc., are of great importance [67,69,76,79]. Collop et al. [57] implemented Burgers analogical constitutive model into FEM to predict viscoplastic deformation. A comprehensive FEM study by [80] investigated the effect of loading scenario in 2D and 3D modeling spaces and implemented the PANDA constitutive law. They investigated 11 different loading modes (given in Table 4) and compared three different material constitutive equations: elasto-viscoplastic, viscoelastic–viscoplastic, and viscoelastic–viscoplastic–viscodamage or PANDA. Based on wheel tracking test data, they concluded that the 2D model significantly overestimates permanent deformation (rutting) compared to the 3D moving load case (which is the most realistic case). Moreover, they found that the viscoelastic–viscoplastic–viscodamage model gives higher rutting damage predictions compared to elasto-viscoplastic and viscoelastic–viscoplastic models. The viscoelastic–viscoplastic and elasto-viscoplastic models give close predictions, where the form gives a still higher rutting prediction. On the contrary, another study using the viscoelastic–viscoplastic–viscodamage constitutive model and 3D FEM modeling of tire–pavement interaction showed that the pulsatile and equivalent loading assumptions overestimated rutting compared to the realistic moving load [90]. The FEM simulation has been applied for different viscoelastic, viscoplastic, and crack modeling. The linear viscoelastic simulation has been modeled using the analogical models (e.g., Maxwell model), the Prony series, and time–temperature superposition principles in commercial software such as ABAQUS.

## 5. Permanent Deformation and Fatigue Interaction Damage

Permanent deformation and fatigue cracking are the two dominant pavement damage mechanisms. Extensive research has been conducted to characterize the two damages independently. Advanced mechanistic models were proposed [64,83,84,91]. Fatigue damage occurred due to the formation and propagation of cracks due to repetitive loads. The bottom-up cracking of the asphalt concrete layer was the traditional fatigue mechanism. However, experimental and field observations showed that top-down cracking due to tire compression is also the cause of fatigue damage [92,93]. The crack initiation in compression occurs when the viscoplastic strain hardening reaches saturation at the flow number. The extra energy at maximum saturation (hardening) is consumed to initiate microcracks and increase phase angle. When asphalt with pre-existing cracks is subjected to a compressive load, wing cracks develop and propagate parallel to the load direction [94,95], contrary to tensile loading (cracks grow perpendicular to the stress direction). When pavement temperature is considered, fatigue cracking is critical at low temperatures, and permanent deformation is a high-temperature damage. At elevated temperatures, the critical energy threshold (the mixture relaxes faster) becomes more resistant to micro-cracks and needs more energy to initiate cracks [96]. On the other hand, deformation via plastic flow (aggregate re-orientation) is dominant at high temperatures. When asphalt concrete is subjected to repetitive loading, energy is dissipated by viscous flow and/or plastic flow, leading to *fatigue cracking* and/or *permanent deformation* [97], and some part of the energy is transferred into heat [50]. The energy dissipation caused material ductility exhaustion, hardening, and viscoplastic flow. The classic energy balance principle states that the decreasing rate of potential energy (stored and recoverable) during crack initiation is equal to the dissipated energy rate due to plastic/viscoplastic deformation and crack opening, and different failure criteria were proposed [98,99]. The dissipated energy (W) in a cyclic load is the area under the stress–strain hysteresis and expressed as follows.

(25) $$W=\overset{\tau }{\underset{0}{\int }}\sigma \left(t\right)\frac{\partial \varepsilon \left(\tau \right)}{\partial \tau }d\tau$$

where τ is the integration variable, σ is the stress function, and ε is the strain function. One hysteresis cycle consists of the total strain energy, which is the sum of damage-causing dissipated energy (the hysteresis area) and the recovery strain energy (stored energy).

**Figure 15:**
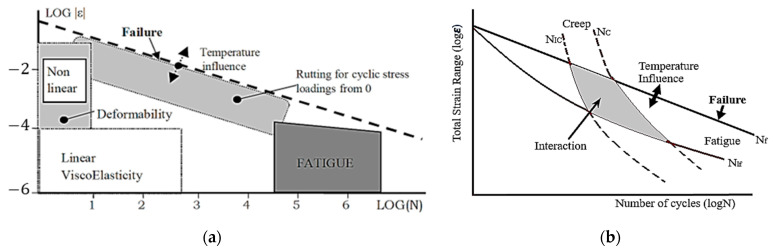
(**a**) Damage domains of bituminous mixtures (ε—strain amplitude and N—number of cycles) and (**b**) fatigue and permanent deformation interactive damage for asphalt concrete (N_C_, N_IC_, N_f_, and N_If_ failure lines for creep and fatigue domains).

The classic approach considered rutting and fatigue separately (cf. Figure 15a) [100]. However, both fatigue and permanent deformation damages are caused by the same load in pavements. The difference is which damage mode dominates at different temperatures. Therefore, a realistic damage modeling should consider the simultaneous damage evolution of fatigue and permanent deformation. An interaction domain of rutting and fatigue can be shown in Figure 15b [101]. For an interaction damage condition, the dissipated energy (in Equation (26)) can be decomposed further into permanent deformation and fatigue damage-causing energies. As discussed above, the existing models for permanent deformation or fatigue damages are based on the idealized hysteresis loops of pure creep and pure fatigue. Only very few attempts can be found; for example, Luo et al. [99] investigated the permanent deformation behavior of pre-cracked asphalt concrete.

**Figure 16:**
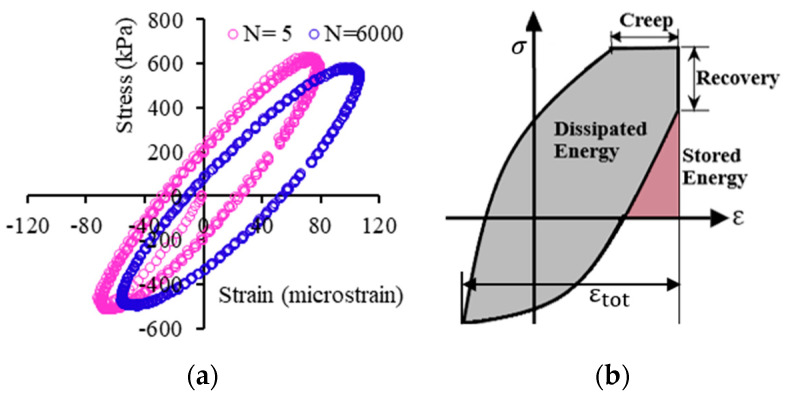
(**a**) Cyclic fatigue hysteresis and (**b**) creep–fatigue interaction stress–strain loop.

In a cyclic creep-recovery test, material hardening grows, and the hysteresis loops shift horizontally due to the accumulation of irrecoverable viscoplastic strain (open loops). On the other hand, in the idealized fatigue test (either stress- or strain-controlled), cyclic load is applied that causes stiffness reduction and a phase angle increment with a negligible viscoplastic strain (hysteresis loops do not shift horizontally or loops are closed). In simultaneous fatigue-permanent deformation damage, the material hardening contributes to initiate fatigue cracking at intermediate temperatures, and the fatigue cracks accelerate the microstructure change and contribute to viscoplastic deformation. The viscous flow creates a plastic zone (the crack initiation point), and fatigue cracking can evolve without plastic flow (aggregate movement and re-orientation). That means the simultaneous occurrence of the two damages cannot be ignored as they are interdependent. Thus, the hysteresis loops can be modified by superposing the two loops (Figure 4a and Figure 16a) to account for the interaction of the two damages, as shown in Figure 16b. Similar fatigue–creep interaction loops can be found elsewhere for steel [102,103]. A detailed discussion of the interaction of the two damages is presented in next sections.

One of the limitations for interaction damage modeling is the lack of integrated testing protocol for permanent deformation and fatigue. Some attempts were made, such as Indirect Tension (IDT) testing for deformation and fracture and applying a haversine loading waveform in fatigue tests [104,105]. The haversine load is used with the assumption that the pulse can be decomposed into pure creep and pure sinusoidal components. However, the creep-recovery behavior cannot be captured with such approaches. In another study, Zhang et al. [106] conducted a destructive dynamic modulus test to simultaneously characterize permanent deformation and fracture properties using 16 different asphalt mixtures. In their work, they successfully modeled viscoelastic, viscoplastic, and viscofracture properties using a new destructive dynamic modulus test. Another reason for the independent treatment of fatigue and rutting damages on asphalt concrete damage is the perception that permanent deformation (creep) is expected in the early life of pavements, while fatigue is high-cycle damage after asphalt concrete accumulates sufficient hardening and age. However, this precedence is dependent on several factors such as temperature, loading range (loading time, rest period, deviatoric stress), material stiffness, number of cycles, and other climatic factors. For example, fatigue cracking can evolve before creep damage in stiff and thick pavements. The top-down cracking due to tire compression can develop without permanent deformation. Some field and laboratory observations have also shown that fatigue cracking accompanied rutting [92]. Therefore, both the creep–fatigue and fatigue–creep damage sequences can occur in asphalt concrete pavements [99].

The main objective in the design and service life estimation of asphalt concrete is to estimate the number of cycles ($N_{f}$) to initiate fatigue cracking and/or the critical strain ($\varepsilon_{c}$—rupture strain) that causes the flow or rutting of asphalt concrete. The simplest way to evaluate the interaction damage of creep and fatigue is separately calculating the creep and fatigue damages and adding them together.

(26) $$\sum \frac{n}{N_{f}}+\sum \frac{\varepsilon }{\varepsilon_{c}}=1$$

By the life fraction rule, the sum of fatigue damage ($\phi_{f}$) and creep damage ($\phi_{c}$) equals a certain damage density value, ϕ. The nonlinear summative decomposition of damage can be expressed as follows for a more general representation.

(27) $$d\phi =d\phi_{c}+d\phi_{f}=f_{c}\left(\varepsilon ,\sigma ,\;T,\phi_{c}+\phi_{f}\right){\mathrm{dN}}_{c}+f_{f}\left(\varepsilon ,\sigma ,\;T,\phi_{c}+\phi_{f}\right){\mathrm{dN}}_{f}$$

The classic continuum damage models were used to define the damage rates for creep [63,65] and fatigue damage [107,108]. Lemaitre et al. [109] proposed sequential damage interaction, and the total damage accumulation during one cycle of creep followed by fatigue and fatigue followed by a creep sequence can be expressed as follows (respectively).

(28) $$\phi_{c\_f}=\int_{0}^{n_{c}}\frac{\delta \phi_{c}}{\delta N}\delta N\;+\int_{n_{c}}^{n_{c}+n_{f}}\frac{\delta \phi_{f}}{\delta N}\delta N$$

(29) $$\phi_{f\_c}=\int_{0}^{n_{f}}\frac{\delta \phi_{c}}{\delta N}\delta N\;+\int_{n_{f}}^{n_{f}+n_{c}}\frac{\delta \phi_{f}}{\delta N}\delta N$$

For time-dependent materials such as asphalt concrete, damage densities $\phi_{c\_f}$ and $\phi_{f\_c}$ will not be the same for the same number of cycles in both sequences. This is because of the different modes of damage formation in creep and fatigue and the possible interactive damage one on another. A parameter can be defined for “interactive” damage. Skelton et al. [101] presented analytical expressions to allow creep to be modified by fatigue and fatigue to be modified by creep. The combined equation for the “creep–fatigue” and “fatigue–creep” interactive damages can take the following form.

(30) $$\frac{\phi_{f}}{1-I_{cf}\phi_{c}}+\frac{\phi_{c}}{1-I_{fc}\phi_{f}}=1$$

The interaction coefficients $I_{\mathrm{cf}}$ (creep on fatigue) and $I_{\mathrm{fc}}$ (fatigue on creep) can take any value between zero and unity. The damage density for fatigue and creep can be determined by rearranging terms and solving a quadratic solution. In the creep–fatigue sequence, it is assumed that pure creep is followed by fatigue damage. The fatigue damage part is modified to account for the pre-existing creep damage. Therefore, the creep–fatigue and fatigue–creep interactive damages can be expressed as follows, respectively.

(31) $$\phi_{c}+\frac{\phi_{f}}{1-I_{cf}\phi_{c}}=1,\phi_{f}+\frac{\phi_{c}}{1-I_{fc}\phi_{f}}=1$$

Re-arranging and solving for $\phi_{c}$ and $\phi_{f}$ gives the following expressions.

(32) $$\phi_{c}=\frac{\left(1+I_{\mathrm{cf}}\right)-\sqrt{{\left(I_{\mathrm{cf}}-1\right)}^{2}+4I_{\mathrm{cf}}\phi_{f}}}{2I_{\mathrm{cf}}},\;\phi_{f}=\frac{\left(1+I_{\mathrm{fc}}\right)-\sqrt{{\left(I_{\mathrm{fc}}-1\right)}^{2}+4I_{\mathrm{fc}}\phi_{c}}}{2I_{\mathrm{fc}}}$$

**Figure 17:**
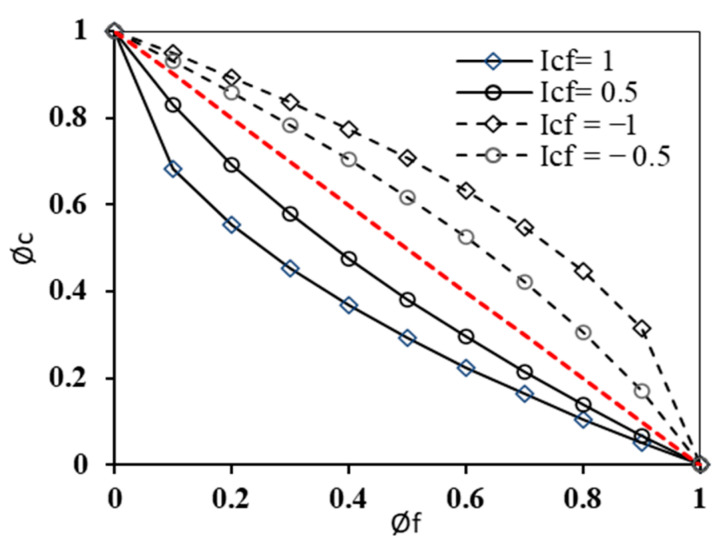
Parametric study of creep–fatigue damage interaction coefficient.

The creep–fatigue and fatigue–creep interaction coefficients $I_{\mathrm{cf}}$ and $I_{\mathrm{fc}}$ are non-zero and take any value between −1 and 1. Figure 17 shows the relationship between creep and fatigue damage densities at random values of creep–fatigue interaction coefficients.

## 6. Summary, Conclusions, Further Research

### 6.1. Summary

The practice is always trailing the theory in pavement design, and the case of the permanent deformation prediction is no different. Many highway agencies and road administrations are practicing an empirical method of permanent deformation characterizations (for example, the Marshall test). Some progresses are being made to transition from empirical to mechanistic-empirical design methods. The primary motivation of this literature study was to explore the advancement of asphalt concrete permanent deformation characterization, constitutive modeling, and application. The current state of research is focused on the formulation of a mechanistic method that applies the fundamental theories of mechanics and materials to predict permanent deformation damage. In the last decade, promising advancements have been made in the development of comprehensive and coupled permanent deformation modeling. Pavement analysis using the nonlinear damage approach (also known as PANDA) constitutive model is one of the notable progresses that is gaining wide acceptance (as the next generation mechanistic pavement design approach). Another promising area of progress is the microstructural approach aided with 3D digital image analysis equipment and finite element modeling. This digital technology-based permanent deformation modeling is also the future prospect for accurate permanent deformation modeling and prediction of different asphalt mixtures. The mechanistic methods offer unlimited potential to expand the modeling parameters, and different damages and phenomena such as aging, healing, moisture damage, pre-crack, etc., can be coupled to unify damage prediction. The drawbacks of mechanistic models are sophistication, requiring extensive testing, and the calibration of several modeling variables. For example, the viscoelastic–viscoplastic–viscodamage model requires more than 21 model variables to be optimized using at least two different experiments and several test repetitions at different temperatures stresses and strains. The improvement of the latest models from the classic viscoplastic strain hardening model is the consideration of cyclic hardening and relaxation mechanisms and the viscodamage of asphalt concrete. Although the mechanistic method is theoretically appealing, the calibration cost and rigorous equations can be considered the limitations. The micromechanics approach considers the evolution of permanent deformation related to changes in the microstructure of asphalt concrete constituents (aggregates and mastic). Thus, the micromechanics method is regarded as the most realistic way of modeling heterogeneous materials such as asphalt concrete. Based on the extensive literature study, the permanent deformation prediction and modeling approaches can be categorized into four aspects, as shown in Table 5.

### 6.2. Conclusions

A review study was conducted to explore the state of the art on permanent deformation prediction from the 1960s’ pure empirical to latest mechanistic (from 2011) methods. The review study revealed that the latest constitutive models integrate and couple different theories, i.e., continuum mechanics (1958), nonlinear viscoelastic (1969), viscoplasticity (1971), and viscodamage (2011), along with the crucial time–temperature superposition principle. Such coupling techniques offered advantages of integrating different asphalt concrete damages and opened the possibility of a unified asphalt damage model in the future. The PANDA model is one of the most comprehensive permanent deformation modeling approaches available in the literature (the next generation to the mechanistic-empirical method). The calibration and/or validation tests are reliant upon the conventional creep or creep-recovery tests in either confined or unconfined modes. The computation and experiment cost of mechanistic methods are the limitations. The practical application of the mechanistic models is very limited at the moment. Moreover, the fatigue–permanent deformation (rutting) interaction is often ignored in the existing (studied) literature. It is inferred that both damages can evolve simultaneously as the same load caused both damages. The mechanistic approach has the potential to couple the two predominant pavement damages. From the extensive study, it can be synthesized that a unified permanent deformation damage model can be developed by integrating continuum damage and microstructure approaches and coupling fatigue, moisture, healing, aging, and other physical and chemical phenomena in asphalt concrete.

### 6.3. Further Research

The mechanistic method is “universally” applicable to predict damage regardless of climatic conditions, stress state, or material type. This characteristic presents wide, open research questions, for example, (1) developing a unified pavement damage performance prediction model, (2) a coupled model for fatigue and rutting damages using continuum mechanics and viscoelastic and viscoplastic theories, (3) considering (coupling) different strains such as shear and axial strains in permanent deformation prediction models, and (4) developing simplified (unified) asphalt concrete test methods to characterized different damages simultaneously (fatigue, rutting, moisture, etc.). The authors of this paper are conducting research on the simultaneous creep–fatigue damage evolution in a sequential manner.

## Figures and Tables

**Table 2:** Summary of material parameters of the constitutive equations and physical meaning in Figure 10.

Parameter	Physical Meaning	Theory/Domain	Calibration Test
*Viscoelastic Model Parameters* [68,82]			
$D_{o}$	Instantaneous creep compliance. Characterizes the instantaneous elastic part of the viscoelastic strain	Linear viscoelastic	Creep compliance or Dynamic Modulus
$\lambda_{n}$	nth retardation time. A measure of the required time for the viscoelastic material to relax the induced stress		
$D_{n}$	nth coefficient of the Prony series associated with the nth retardation time kn. These parameters characterize the transient compliance of the material		
$g_{o}$, $g_{1}$, $g_{2}$	Nonlinear viscoelastic parameters, where $g_{o}$ measures the reduction or the increase in the instantaneous compliance; $g_{1}$ defines the nonlinearity effects in the transient compliance; and $g_{2}$ is the nonlinear parameter accounting for the loading rate effects on the creep response	Schapery’s Nonlinear viscoelastic	Creep-recovery
*Viscoplastic Model Parameters* [64,66,69]			
$\Gamma^{vp}$	Viscoplastic fluidity parameter, such that $1/\Gamma^{vp}\;$ is a measure of viscoplastic viscosity	Perzyna’s Viscoplastic (with Drucker–Prager yield criteria)	Creep, creep-recovery (creep part), constrain strain rate test
$\kappa_{o}$, $\kappa_{1}$, $\kappa_{2}$	Isotropic hardening parameters, where $\kappa_{o}$ defines the initial yield strength; $\kappa_{o}+\kappa_{1}$ defines the saturated limit of the hardening function; and $\kappa_{2}$ defines the hardening rate and controls the shape of the hardening function versus the effective viscoplastic strain (${\dot{\varepsilon }}_{e}^{vp}$)		
N	Viscoplastic rate sensitivity exponent and describes the nonlinear rate dependency of viscoplastic response		
α , β	Govern the pressure sensitivity of the yield surface and plastic potential functions. Related to the angle of friction in the asphalt concrete		
$d^{\mathrm{vp}}$	Model parameter distinguishing viscoplastic responses in extension and contraction modes of loading		
*Visco-damage Model Parameters* [64]			
$\Gamma^{vd}$	Visco-damage fluidity parameter ($1/\Gamma^{vd}$ is a measure of damage viscosity)	CDM (Tertiary creep)	Creep tests
q	Stress dependency parameter. Defines the sensitivity of the damage evolution due to stress level		
$Y_{o}$	The reference damage force obtained at a reference stress of a creep test		
k	Strain exponent parameter. Defines the sensitivity of the damage evolution due to strain level		
$d^{\mathrm{vd}}$	Model parameter distinguishing visco-damage responses in extension and contraction modes of loading		
*Micro-damage healing model Parameters* [83]			
$\Gamma^{h}$	Micro-damage healing fluidity parameter, such that $1/\Gamma^{h}$ is a measure of healing viscosity	CDM for healing	
$k_{1}$, $k_{2}$	Healing model parameters that describe the effect of the damage and healing histories on the healing evolution		
*Temperature coupling terms parameters* [64]			
$\theta_{1}$, $\theta_{2}$, $\theta_{3}$	Temperature sensitivity model parameters for viscoplastic, viscodamage and microdamage healing, respectively	Time–temperature superposition	Dynamic Modulus, Creep compliance
$T_{o}$	Reference temperature		
*Hardening–relaxation Model Parameters* [24,25]			
$\Gamma^{h-r}$	The hardening–relaxation fluidity parameter, such that 1/$\Gamma^{h-r}$ is the hardening–relaxation retardation time controlling the rate of the hardening–relaxation		Creep-recovery (at different rest periods)
$S_{1}$, $S_{2}$, $S_{3}$	Hardening–relaxation rate-sensitivity parameters that describe the relaxation behavior of viscoplastic hardening due to recoverable potential during rest period		
*Moisture damage Model Parameters* [84]			
p	Adhesion or cohesion moisture damage parameter	CDM for moisture damage	Pull-off test
δ	Parameter that describes the moisture damage history		

**Table 3:** Advantages and limitations of the PANDA Model.

Advantages	Disadvantages
▪ The approach is based on fundamental theories of mechanics (mechanistic) ▪ Unlimited modeling capacity ▪ The material properties and temperature coupling are integrated ▪ The approach has the capacity to couple different damage types (such as moisture, fatigue cracking, etc.) with permanent deformation ▪ It enables realistic rutting prediction with full pavement structure modeling using finite element method (3D modeling) ▪ Moving loads can be modeled, which was not possible in traditional methods etc.	▪ Complex constitutive equations ▪ Large number of modeling parameters ▪ It requires many different calibration tests ▪ It is computationally and experimentally costly ▪ It is at development stage and some theories have limitations (e.g., classic viscoplasticity theory) ▪ The numerical implementation is based on user material (UMAT) model (not standalone) ▪ etc.

**Table 4:** The commonly used FEM simulation loading assumptions.

		Loading Approach		
Mode	Load Configuration	Pulse Loading	Equivalent Loading	Moving Loading
2D	Single wheel (plane strain)	√	√	√
	Single wheel (axisymmetric)	√	√	√
	Single wheel (moving loading)			√
	Multiple wheel (moving loading)			√
3D	Single wheel (rectangular)	√	√	√
	Whole wheel path	√	√	
	Single wheel (circular)	√	√	
	Single wheel (moving)			√
	Multiple wheel			√

^√^ FEM simulation applied.

**Table 5:** Summary of permanent deformation modeling approaches.

	Approach			
Properties, Theories, Methods	PANDA	Microstructure	Analogical	Empirical
Continuum damage mechanics	√	√	-	-
Viscoelastic	√	-	√	√
Viscoplasticity	√	√	√	√
Micromechanics	†	√	-	-
Finite element simulation	√	√	√	-
Time–temperature superposition	√	√	√	-
Coupling (healing, moisture)	√	√	-	-
Coupling fatigue–rutting damage	-	-	-	-
Full pavement deformation model	√	√	-	-
Hardening–relaxation mechanism	√	√	-	-
Uniaxial and triaxial repeated load test	√	√	√	√

√ Incorporated, † Not yet incorporated.

## Data Availability

Not applicable.
